# Effective immuno-targeting of the IDH1 mutation R132H in a murine model of intracranial glioma

**DOI:** 10.1186/s40478-014-0180-0

**Published:** 2015-01-21

**Authors:** Serena Pellegatta, Lorella Valletta, Cristina Corbetta, Monica Patanè, Ileana Zucca, Federico Riccardi Sirtori, Maria Grazia Bruzzone, Gianpaolo Fogliatto, Antonella Isacchi, Bianca Pollo, Gaetano Finocchiaro

**Affiliations:** Department of Neuro-Oncology, Unit of Molecular Neuro-Oncology, Fondazione I.R.C.C.S. Istituto Neurologico C. Besta, via Celoria 11, 20133 Milano, Italy; Nerviano Medical Sciences, Nerviano S.r.l., Milano, Italy

**Keywords:** IDH1-R132H, Immunotherapy, Glioma, 2-hydroxyglutarate

## Abstract

The R132H mutation of cytosolic isocitrate dehydrogenase (IDH1) is present in the majority of low grade gliomas. Immunotherapy in these tumors has an interesting, still unexploited, therapeutic potential, as they are less immunosuppressive than glioblastomas.

Using site-directed mutagenesis we introduced the R132H mutation into the murine glioma cell line GL261, creating mIDH1-GL261. Presence of the mutation was confirmed by immunoblotting and production of the oncometabolite 2-hydroxyglutarate (2HG), demonstrated by mass spectrometry (LC-MS/MS) performed on cell supernatant. In vitro mIDH1-GL261 had different morphology but similar growth rate than parental GL261 (p-GL261). After intracranial injection, MRI suggested that the initial growth rate was slower in mIDH1-GL261 than p-GL261 gliomas but overall survival was similar. mIDH1-GL261 gliomas showed evidence of R132H expression and of intratumoral 2HG production (evaluated by MRS and LC-MS/MS). Immunizations were performed nine days after intracranial implantation of mIDH1- or p-GL261 cells by three subcutaneous injections of five different peptides encompassing the IDH1 mutation site, all emulsified with Montanide ISA-51, in association with GM-CSF. Control mice were injected with four ovalbumin peptides or vehicle. Mice with mIDH1-GL261 but not p-GL261 gliomas treated with mIDH1 peptides survived longer than controls; 25% of them were cured. Immunized mice showed higher amounts of peripheral CD8+ T cells, higher production of IFN-γ, and evidence of anti-mIDH1 antibodies. Immunizations led to intratumoral up-regulation of IFN-γ, granzyme-b and perforin-1 and down-regulation of TGF-β2 and IL-10.

These results support the translational potential of immunotherapeutic targeting of gliomas carrying IDH1 mutations.

**Electronic supplementary material:**

The online version of this article (doi:10.1186/s40478-014-0180-0) contains supplementary material, which is available to authorized users.

## Introduction

The new era of cancer genomics, due to the revolution of next generation sequencing, was heralded in 2008 by the discovery of mutations in the IDH1 gene in glioblastomas (GBM) [1]. Subsequently it was found that IDH1 mutations are present in the large majority of low grade gliomas (LGG) and define secondary GBM more rigorously than before [2]. Remarkably, a new function of IDH1 was found as a consequence of the R132H mutation that affects the active site of the enzyme [3]. IDH1, that normally catalyzes the production of alpha-ketoglutarate (alpha-KG), in its mutated form released 2-hydroxyglutarate (2HG), subsequently defined as an oncometabolite [3]. Mutations of the cytosolic IDH1, as well as of the mitochondrial isoform IDH2, were subsequently found in other tumors like cholangiocarcinomas [4], leukemias [5] and others, reviewed in [6]. In gliomas, one main consequence of the R132H mutation is the alteration of the epigenetic patterns, as 2HG may act as a competitive inhibitor of histone and DNA demethylases requiring alpha-KG as a cofactor [7-9]. Initially, increased neo-angiogenesis due to increased expression of the pro-angiogenic factor HIF-1α was also found as a consequence of IDH1 mutation [10].

Due to mutation frequency and importance of the modulation of glioma biology, an increasing effort has been placed on pharmacological targeting of mutated IDH1. New drugs that are acting on the novel function of IDH1 are under development and are initially tested in patients [11]. Furthermore, the diagnosis of the mutation in peripheral blood as well as the detection of increased levels of 2HG by MRI in the brain are actively pursued and initial results are available [12,13].

As in the case of EGFRvIII, the R132H mutation creates a novel, cancer-specific epitope, that could be potentially targeted by immunotherapy, as recently shown by Schumacher et al. [14]. This could be particularly effective in LGG as these tumors, due to their association with longer survival than GBM [15] and with a less immunosuppressive microenvironment [16], may offer a wider window for the development of a therapeutically meaningful immune response. To this goal we have developed a glioma model of the R132H mutation that is immunologically actionable. Our results suggest that peptide vaccination may delay and, in a fraction of cases, cure an intracranial glioma that is otherwise lethal in about one month.

## Materials and methods

### Generation of GL261-IDH1-R132H cell line

Site directed mutagenesis was performed on pCMV6kan-neo vector containing the murine wild-type IDH1 cDNA (NM_010497.3) (Origene, Rockville, MD, USA), human IDH1mutation R132H was introduced (see Additional file 1).

GL261 adherent cells were seeded in 6-well plates at 2 × 10^6^ cells per well 24 hours prior to the transfection. Cells were transfected with 10 μg of empty or IDH1-R132H– DNA vector using Lipofectamine 2000 Transfection Reagent (LifeTechnologies), according to the manufacturer’s instructions. Twenty-four hours after transfection, GL261 cells were split and grown in fresh medium containing 0.5 mg/ml of G418 (Lonza) as selective agent.

Parental and IDH1-GL261 were grown in DMEM, 1X Pen Strep,1X L-glu, 20% Fetal Bovine Serum (LifeTechnologies). The cell Proliferation Reagent WST-1 (Roche) was used to measure proliferation plating 3000 cells/well. Six replicates per point were analyzed.

### Peptide prediction

Mice were immunized with two 9-mer (PEP1 - IDH1-R132H_126–134_: KPIIIGHHA; PEP2 - IDH1-R132H_128–136_: IIIGHHAYG), two 10-mer (PEP3 - IDH1-R132H_131–140_: IGHHAYGDQY; PEP4 - IDH1-R132H_123–132_: GWVKPIIIGH) and a 16-mer (PEP5 - IDH1-R132H_126–141_: KPIIIGHHAYGDQYRA) peptides spanning the IDH1 mutation. Negative control peptides were: OVA1_55–62_: KVVRFDKL; OVA2_107–114_: AEERYPIL; OVA3_176–183_: NAIVFKGL, synthesized by Primm srl (Milano), and OVA4_257–264_: SIINFEKL (Sigma Aldrich).

### In vivo treatments

Five week-old females C57BL/6N mice (Charles River Laboratory, Calco-Lecco Italy) were injected with 50,000 pGL261 cells (n = 44) or mIDH1-GL261 (n = 80). A total of 24 mIDH1-GL261 glioma bearing mice were sacrificed after second immunization for immunological and histological studies (6 mice per group of treatment). The stereotactic coordinates with respect to the bregma were: 0.7 mm posterior, 3 mm left lateral, and 3.5 mm deep, into the nucleus caudatum. Glioma-bearing mice were treated using four different conditions: group I: vehicle only (GM-CSF and Montanide); group II: three immunizations spaced one week apart (days 9, 16 and 23) with two 9-mer and two 10-mer peptides; group III: three immunization spaced one week apart (days 9, 16 and 23) with the 16-mer peptide; group IV: ova peptide immunizations. Single peptides (15 μg/peptide) were emulsified with Montanide ISA-51 VG (1:1; SEPPIC) as previously described [17,18], and administered by subcutaneous injections of the 16-mer peptide (group III) or the four peptides separately (group II and group IV) into different areas of the flank. The immunized mice received a total of 3 μg of recombinant murine granulocyte macrophage colony-stimulating factor (GM-CSF) (Miltenyi Biotec) spread out over three injections into the same area of the peptide injections, beginning one day before the first vaccination.

Animal experiments were performed in accordance to the Italian Principle of Laboratory Animal Care (D. Lgs. 26/2014) and European Communities Council Directives (86/609/EEC and 2010/63/UE).

### Isolation and characterization of splenocytes

Splenocytes from immunized and control mice were isolated after the second immunization. Before staining, splenocytes were grown in RPMI 1640 (EuroClone) containing 10% heat-inactivated fetal bovine serum (FBS) (Life Technologies), 100 U/mL penicillin (EuroClone), 100 U/mL streptomycin (EuroClone), 100 μg/mL glutamine (EuroClone), 0.1 mM non-essential amino acids (EuroClone), 1 mM sodium pyruvate (EuroClone), 50 μM β-mercaptoethanol (EuroClone) and 10 U/mL IL-2 (Miltenyi Biotec). Cells were stimulated for 4 h with 0.25 μM ionomycin and 10 ng/mL phorbol myristate acetate (PMA) and 2 hours with 10 μg/ml brefeldin A. A total of 1.5 × 10^6^ cells were stained in PBS 1X/0.5% bovine serum albumin/2 mM EDTA for 10 min at 4°C with the following antibodies (Miltenyi Biotec): anti-CD3 Vioblue; anti-CD4-APC, anti-CD8-PE-Vio770, anti-CD62L-FITC and anti-CD49d-PE for T cell detection. The cells were then fixed and permealized using the Miltenyi Biotec Inside Stain Kit and intracellularly stained according to the manufacturer’s instructions with IFN-γ-PE or APC (Miltenyi Biotec). The CD8 + CD3+; CD3 + CD4+ cells were gated and then analyzed by flow cytometry for IFN-γ production and for the evaluation of CD62L and CD49d expression. Flow cytometry acquisition was performed on a MACSQuant instrument, and the data were analyzed with the MACSQuantify Software (Miltenyi Biotec).

### Cytotoxic and proliferation assays

Lymphocytes from immunized and control (vehicle and ova-treated) mice were isolated after second immunization. A total of 2 × 10^6^ splenocytes were pre-stimulated for 5 days in the presence of 5 × 10^5^ irradiated (3-Gy), naïve splenocytes, acting as antigen presenting cells, and 10 μg/mL of each peptide. Pre-stimulated splenocytes were tested for GL261-specific cytotoxicity using 10:1, 20:1 and 40:1 effector:target (E:T) ratios. The cytotoxic MTT assay was performed according to the manufacturer’s instructions (Millipore). For proliferation assay, 2 × 10^6^ splenocytes were primed for 4 days in the presence of 5 × 10^5^ naïve splenocytes that had received 3 Gy irradiation, 10 μg/mL of IDH1 mutated or OVA peptides and 10 U/ml of IL-2. After pre-stimulation, 5 × 10^5^ splenocytes were incubated for 20 h in the presence of single peptides (10 μg/ml) and 10 U/ml IL-2 and tested for their ability to proliferate using MTT Reagent (Millipore). The data are expressed as the percentage of proliferation, calculated according to the following equation: (OD stimulated splenocytes – OD splenocytes without peptide)/OD stimulated splenocytes × 100.

### IgG ELISA

ELISA plates were coated with 0.01 mg/ml IDH1 R132H peptides for IgG detection in sera of immunized and control mice. Ovalbumin peptides were used as peptide coating control. The plate was washed and blocked with PBS 10% FCS for 2 h at room temperature. The plate was washed and mouse sera (1:100) were incubated for 2 h at room temperature. After washing the plate, HRP-conjugate secondary antibody IgG-HRP (1:5000) was incubated for 1 h in the dark at room temperature. The plate was washed and incubate with tetramethylbenzidine (TMB) and the reaction was stopped with H_2_SO_4_ 1 M. OD at 450 nm was measured.

### Real-time PCR (RT-PCR)

Total RNA was isolated from freshly harvested GL261 gliomas and lymphocytes from immunized and control mice and used for gene expression analysis. RNA was extracted with TRIzol reagent (Life Technologies) using the RNeasy MINI KIT (Qiagen) and the RNase-Free DNase Set (Qiagen). cDNA was synthesized from total RNA using oligo (dT) and M-MLV Reverse Transcriptase (Life Technologies). Specific primers for target genes were designed for Fast SYBR Green chemistry (Life Technologies) and purchased from Primm S.r.l. The relative mRNA levels were evaluated using a ViiA-7 Real-Time PCR System (Life Technologies) and calculated using the ΔΔCt method. The expression levels of the target genes were normalized to the expression level of beta-actin (see oligo sequences in Additional file 1).

### Western blot

Total proteins were isolated using tissue lysis with PK buffer (NaH_2_PO_4_ 0.2 M; Na_2_HPO_4_ 0.2 M; NaCl 5 M; EDTA) and protease inhibitor buffer (Triton 100X 2%; SDS 0.25%; Leupeptin 0.90%; Pepstatin A 0.90%; PMSF 0.90%), and centrifuged to pellet debris. Protein concentrations were measured by Micro BCA protein assay kit (Thermo Scientific) at 540 nm. Proteins (20 μg) were diluted in NuPAGE® LDS Sample Buffer 4X (Invitrogen) and NuPAGE Reducing Agent 10X (Invitrogen) and electroblotted onto nitrocellulose membranes at 30 Volts for 1 h . Membranes with transferred proteins were incubated with the primary antibody anti-IDH1-R132H (1:100, Dianova) or anti-vinculin (1:5000, Abcam, Cambridge, UK). The primary antibody incubation was followed by incubation with the secondary antibody anti-mouse (1:10000). A chemiluminescence reaction using the ECL (enhanced chemiluminescence) Plus kit (Amersham, GE Healthcare) was detected using film.

### Liquid chromatography (LC-MS/MS) and magnetic resonance spectroscopy

Tumour lysates (100 μL) were denatured by adding 10 μL of thrichloroacetic acid (TCA) 1 M containing 2HG-d3 (130 μM) as internal standard. After mixing and centrifugation, the supernatants were analyzed for 2-hydroxyglutaric (2HG) determination using an Ultra High Pressure Liquid Chromatography system (UPLC®, Waters) coupled with a triple quadrupoles mass spectrometer (TQD, Waters) operating in multiple reaction monitoring mode (MRM). Protein normalization was obtained using the microBCA kit (Thermo Scientific). Supernatants were obtained from a sub-confluent cell cultures (3 × 10^6^ cells).

MRI/MRS studies were performed on a 7 Tesla BioSpec 70/30 USR scanner equipped with a 12 cm inner diameter actively shielded gradient system reaching a maximum amplitude of 400 mT/m. (Bruker BioSpin, Ettlingen, Germany). T2-weighted images were acquired in three orthogonal (axial, sagittal, and coronal planes) using a rapid acquisition with relaxation enhancement (RARE) sequence (TR/TE = 3000/13 ms, matrix size = 256 × 256, RARE factor = 8, slice thickness = 0.7 mm, FOV = 2 × 2 cm2, in plane resolution = 78 μm, number of averages(NA) = 1, acquisition time (AT) = 1 min 30 s). 1H spectra carried out by two PRESS sequence with different Echo Time (TE) TE =13 ms and 50 ms (Point RESolved Spectroscopy, TR = 5000 ms, and adjustment of the first and second order shims conducted beforehand by Mapshim macro of Paravision 5.1 software) from a single voxel (~10 μL) located inside a tumor (or, before detecting solid tumor, at the injection site). The spectra were analyzed with LCModel software (Version 6.3) [19] for metabolites absolute quantification. The metabolites concentration for each single mouse at different time point (before, one week, twenty days and one month after the tumor cells injection) are presented as absolute concentration ± CRB error [20]. The metabolites with CRB error less than 20% of the estimated metabolite concentration were considered. Also the relative concentration with respect to Creatine is reported.

### Histology and immunohistochemistry

For histology of murine gliomas, brains were carefully removed, post-fixed in 4% paraformaldehyde, and embedded in paraffin. Five μm thick sections were dissected using a microtome. IHC analysis for IDH1-R132H was performed on paraffin-embedded sections using the anti-IDH1-R132H antibody (Dianova). The paraffin was removed using xylene, and the sections were rehydrated in graded alcohol. Antigen retrieval was carried out using preheated target retrieval solution (pH 6.0), and the primary antibodies were incubated overnight. Single immunostains were performed using a standard immunoperoxidase protocol (Vectastain Elite ABC kit, PK-6100; Vector Laboratories, Inc., Burlingame, CA, USA) followed by a diaminobenzidine chromogen reaction (Peroxidase substrate kit, DAB, SK-4100; Vector Lab). All sections were counterstained with Mayer’s hematoxylin and visualized using a LEICA MDLB light microscope.

### Statistical analysis

Statistical comparisons of the data sets were performed using a two-tailed Student’s *T*-test, and the results were considered significant at *p* < 0.05. Cumulative survival curves were constructed using the Kaplan-Meier method (MedCalc 9.3).

## Results

### GL261-cells overexpressing IDH1-R132H produce 2HG and are tumorigenic in vivo

**Figure 1:**
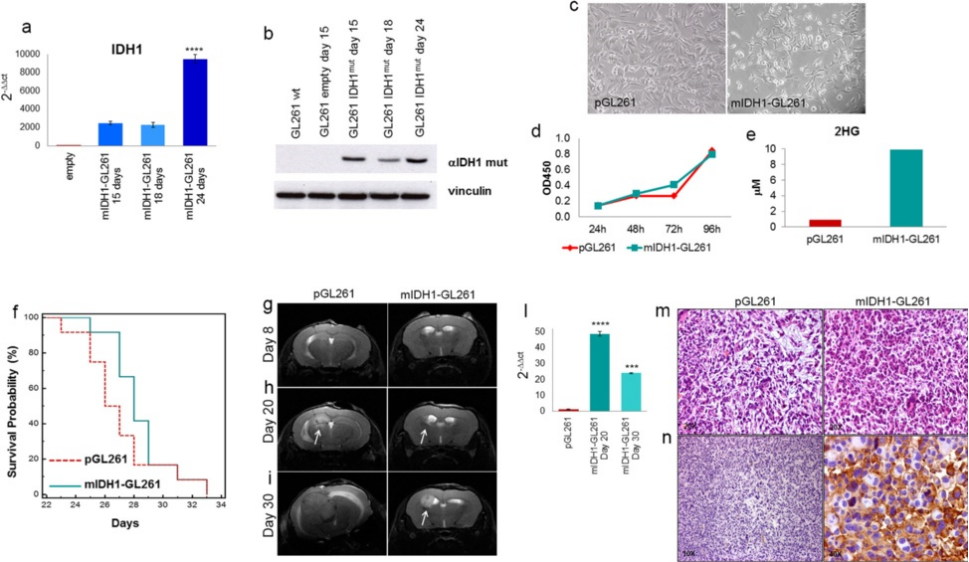
**mIDH1-GL261 cells release 2HG in vitro and are tumorigenic. a** RT-PCR analysis shows that IDH1 expression was significantly higher in mIDH1-GL261 compared to control cells. **b** Western blot analysis of IDH1-R132H expression in transfected GL261 cells after 15, 18 and 24 days of selection. Compared to empty (control cells), the levels of IDH1-R132H are significantly increased. Vinculin was used as housekeeping protein. **c** GL261 cells over-expressing IDH1-R132H selected for 24 days in the presence of G418 changed morphology compared to control cells. **d** Proliferation of pGL261 and mIDHI-GL261 as shown by WST proliferation assay performed at four different time points. **e** mIDH1-GL261 released 2HG in the cell supernatant as evaluated by LC-MS/MS. **f** mIDH1-GL261 when injected in vivo grew slower than control, however in the later stages the survival curves overlapped (n = 8 mice/group, *p* = 0.36). **g, h, i** MRI T2-weighted images confirmed the slower engraftment of mIDH1-GL261 glioma-bearing mice compared with controls at three different times. **l** RT-PCR performed on explanted gliomas shows that IDH1 overexpression was higher that controls at day 20 and begins to decrease 30 days after tumor implantation. **m**, **n** Representative IHC shows the expression of IDH1-R132H in gliomas from mIDH1-GL261 compared to control pGL261-gliomas where expression of IDH1 mutation was totally absent.

To verify the potential effects of IDH1 mutation on tumor progression, we over-expressed IDH1-R132H in GL261 cells creating mIDH1-GL261. Expression of mIDH1 mRNA was detectable by RT-PCR in mIDH1-GL261 15 days after selection with G418, and highly increased on day 24 after transfection (Figure 1a). Accordingly, expression of the mutant protein was clearly observed from day 15 by Western blot with R132H mutation specific antibodies (Figure 1b). At the same time point mIDH1-GL261 cells appeared smaller that pGL261 and with fibroblast-like features (Figure 1c). No significant difference in proliferation was visible in vitro, as evaluated at four time points (24, 48, 72 and 96 h, Figure 1d). mIDH1-GL261 released 2HG in micromolar amounts in the culture medium, as measured by LC-MS/MS (Figure 1e).

The overall survival of mice injected intracranially with mIDH1-GL261 cells appeared initially longer than in pGL261 controls, but the two curves merged later (Figure 1f). Observations on tumor size by MRI were coherent with survival data. Eight days after implantation, in both groups no signal alteration on T2-weighted (wi) images was evident along the injection track (Figure 1g). On day 20 the tumor onset was detected as a bright area on the T2-wi images. pGL261-gliomas were growing more rapidly than mIDH1-gliomas but remained small and circumscribed (Figure 1h). Thirty days after tumor cell injection, the mIDH1-GL261 gliomas were still smaller but brighter than pGL261 gliomas (Figure 1i). By RT-PCR on explanted gliomas we found that the expression of IDH1 was significantly reduced over the time, from 48.8 ± 1.3 on day 20 to 24. ± 0.4 on day 30 (p < 0.001) (Figure 1l).

Histology with H&E staining showed that pGL261-gliomas were characterized by pleomorphic and mitotically active cells, while mIDH1-gliomas were composed of single small and round cells occasionally organized as cell nodules (Figure 1m). Immunostaining with specific antibodies confirmed the expression of IDH1-R132H in gliomas originating from mIDH1-GL261, but not in pGL261 gliomas (Figure 1n).

### mIDH1-GL261 gliomas maintain 2HG production

**Figure 2:**
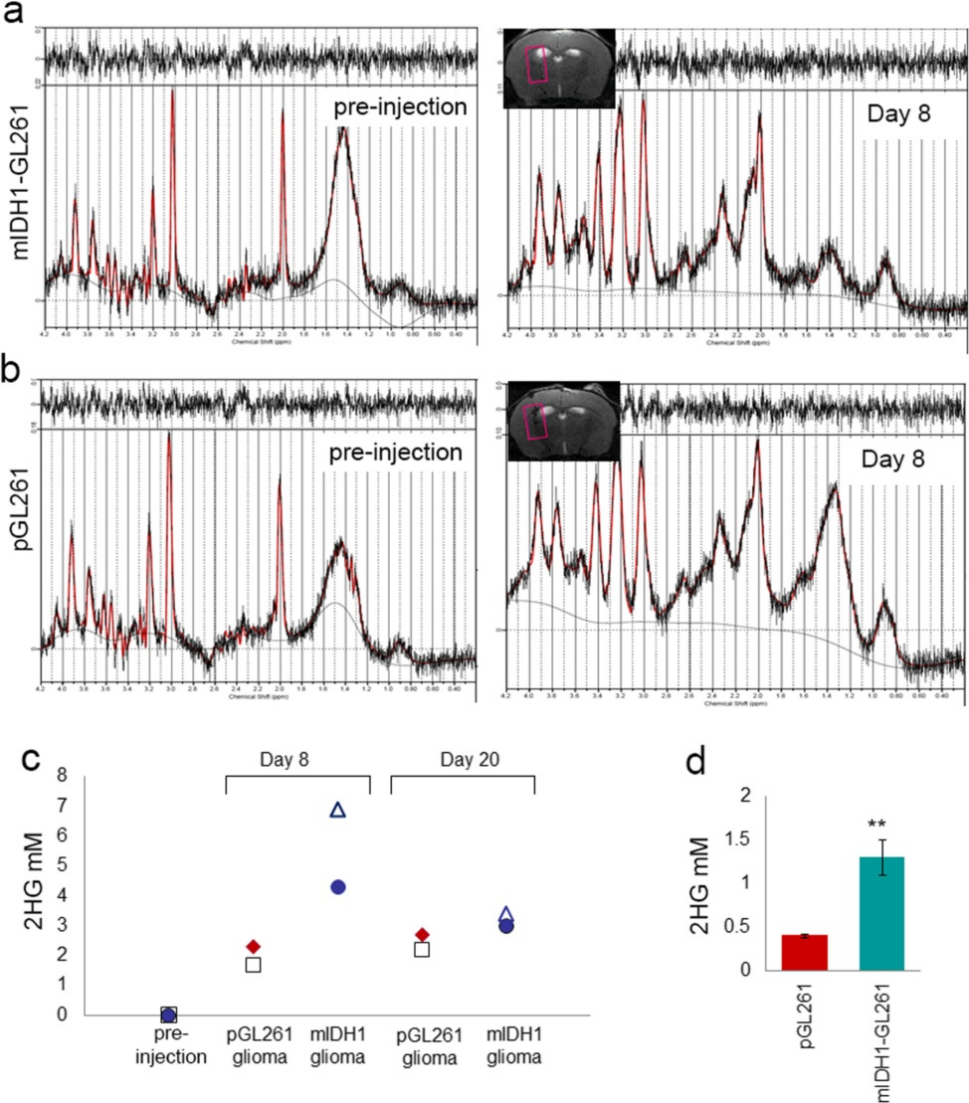
**2HG concentration is higher in mIDH1-GL261 than pGL261-gliomas. a, b** Representative spectra show variation of brain metabolite concentration measured at the injection site of gliomas from mIDH1-GL261 compared to controls. Data were acquired before cell injection (pre-injection), and 8 days after tumor implantation. **c** 2HG concentration increased in mIDH1-GL261 gliomas at day 8 and decreased on day 20 possibly as a consequence of a decreased expression of IDH1 as evaluated by RT-PCR on explanted gliomas (Figure 1l). **d** Intratumoral 2HG measured at day 30 showed higher levels in mIDH1-GL261 than in pGL261-gliomas.

In order to evaluate the absolute concentration of 2HG, magnetic resonance spectroscopy (MRS) was performed on mice before and after (on day 8 and 20) pGL261 or mIDH1-GL261 implantation. A preliminary optimization of 2HG detection was performed by acquiring sequences with two different Echo Time values: TE = 13 ms and TE = 50 ms for each examination session (not shown). A more accurate quantification was obtained using spectra with the shorter TE, as also previously described [21]. In pre-injection MRS data, there was no evidence of 2HG in all the examined animals (Figure 2a and b, left panels; quantification in Figure 2c). The improved signal-to noise ratio and the use of quantitative standards allowed to obtain for the first time an absolute quantification of 2HG in mouse brain. On day 8 mIDH1-GL261 gliomas showed a significantly higher concentration of 2HG as compared to pGL261 gliomas (4–7 mM vs 2 mM, Figure 2c and a, b right panels). Interestingly an accumulation of 2HG higher than the background was also reported in a subgroup of human wild type IDH gliomas [22]. Decreased concentration of 2HG was detected on day 20 (Figure 2c). No difference in the absolute concentration of the other metabolites investigated was observed (e.g. choline, lactate, N-acetyl-aspartate). The levels of 2HG were directly measured by LC-MS/MS in gliomas explanted on day 30, confirming the presence of millimolar levels of the metabolite, with higher expression in the mIDH1-GL261 (Figure 2d).

mIDH1-GL261 gliomas appeared macroscopically more hemorrhagic than pGL261-gliomas and showed increased levels of HIF-1α and VEGF (Additional file 2: Figure S1) [10,23].

### Immunization with mutated IDH1 peptides significantly increases survival inducing specific cytotoxic and antibody responses

**Figure 3:**
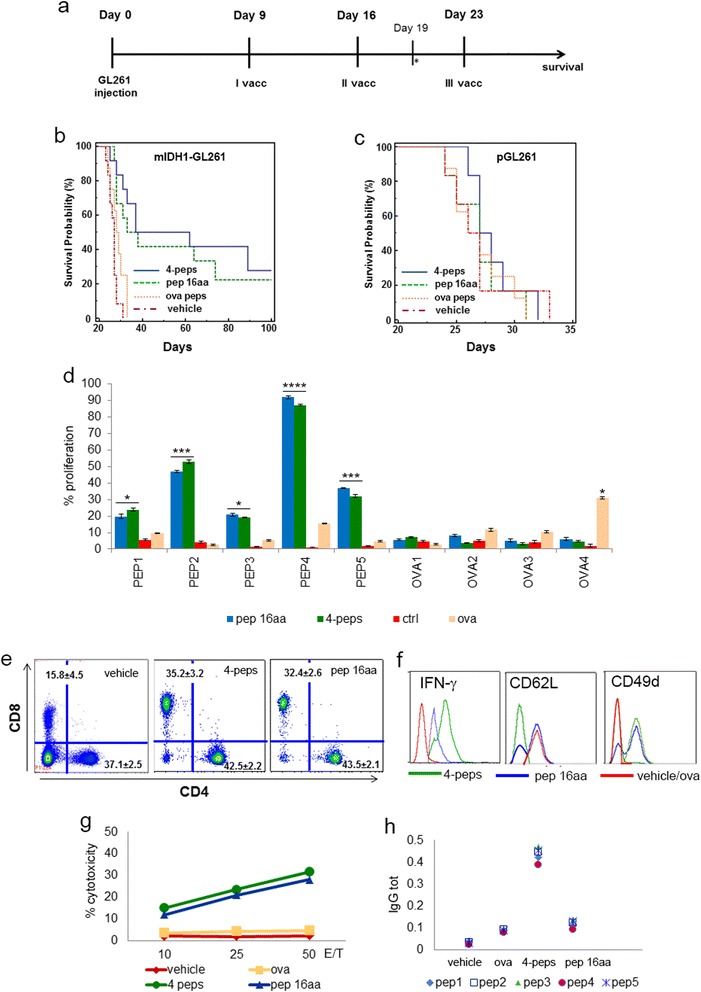
**Immunization with mutated IDH1 peptides prolongs survival and promotes specific anti-tumor responses. a** Experimental schema of in vivo treatments. Two groups or mice injected with mIDH1-GL261 or pGL261 were immunized with three subcutaneous vaccinations on days 9, 16 and 23 after tumor implantation with four peptides (two 9-mer and two 10-mer), or with a single peptide (16-mer) emulsified in Montanide (15 μg/single peptide). A total of 3 μg of GM-CSF/mouse were administered during each immunization. Two group of control mice were treated with GM-CSF and Montanide alone or with ova peptides. On day 19 mice were sacrificed for immune monitoring. **b**, **c** Kaplan-Meier curves show that immunized mIDH1-glioma mice (four peptides n = 12; 16-mer peptide n = 12) survived longer than control mice (vehicle, n = 12; ova n = 12). Immunizations of p-GL261 glioma-bearing mice did not modify the survival. **d** Splenocytes from immunized mice proliferated significantly more than splenocytes from control (vehicle and ova treated) mice in the presence of IDH1-R132H peptides, particularly of peptide 4 (*p < 0.01; **p < 0.005; ***p < 0.001, ****p < 0.0001), not in presence of ova peptides. **e** Flow cytometry performed on splenocytes shows that CD8+ but not CD4+ T cell percentage increased significantly in immunized mice compared to controls (n = 6 mice/group). No difference between vehicle and ova peptide immunizations. Data are reported in dot plots as the mean% ± SD. **f** Flow cytometry histograms show that CD3 + CD8+ T cells produced more IFN-γ and expressed less CD62L and more CD49d than controls (vehicle and ova histograms were overlapped). **g** In vitro MTT cytotoxicity assay reveals that splenocytes from immunized but not from control mice lyse mIDH1-GL261 cells. **h** Scatter plot shows mIDH1 specific IgG detected in serum from mice immunized with four peptides. Single plots represent the mean of three serum samples/group.

To determine whether IDH1-R132H could be a target for glioma immunotherapy we treated mIDH1-GL261 glioma-bearing mice with four short peptides (two 9-mers and two 10-mers) or one 16-mer peptide, all encompassing the IDH1 region encoding the mutation. Vaccinations with peptides were performed on days 9, 16, and 23 after tumor implantation (Figure 3a). Peptide immunizations significantly increased survival as compared with vehicle-treated and ovalbumin (ova) peptide-treated controls (Figure 3b). Immunization of pGL261-glioma-bearing mice using the same peptides was ineffective (Figure 3c), demonstrating that the immune response induced by peptide vaccines specifically targets mIDH1-expressing cells.

Immunogenicity and functionality of peptides used for immunizations was assessed by an in vitro proliferation assay (Figure 3d). Splenocytes from control (vehicle and ova), or from immunized mice were stimulated using a mixture of irradiated antigen presenting cells and peptides. Four days later, single peptides were assessed for the ability in inducing lymphocyte proliferation. Splenocytes from immunized mice proliferated in the presence of IDH- mutated peptides but not of ova peptides. PEP4, in particular, induced 90% of proliferation. On the contrary peptides were unable to stimulate splenocytes from “vehicle” mice. Splenocytes from ova-immunized mice proliferated when in presence of one out of four peptides, confirming the ability of this peptide to induce some cytolytic response [24].

We observed a significant increase of CD8+ T cell response in spleens of immunized mice compared with controls (Figure 3e). CD8+ T cells from mice immunized with four peptides produced higher level of IFN-γ and lost CD62L expression indicating the effector phase of the lymphocytes (Figure 3f). They expressed higher levels of CD49d, critical for efficient infiltration and homing into the glioma (Figure 3f) [25]. CD8+ T cells from mice immunized with the 16-mer peptide showed an intermediate expression of IFN-γ and two subpopulations with high or absent CD62L expression, suggesting the coexistence of both naïve and effector cells (Figure 3f). Part of the splenocytes from immunized mice were pre-stimulated with peptides and tested five days later for cytotoxic ability using an MTT assay. The splenocytes from mice treated with IDH1-R132H peptides displayed specific cytotoxicity against mIDH1-GL261 compared with vehicle and ova controls (Figure 3g), but not against pGL261 (Additional file 2: Figure S2). IDH1-R132H specific IgG were detected in serum of mIDH1-GL261 glioma bearing mice after immunization with the four peptides compared with controls. No IgG response was found after immunization with the single 16-mer peptide (Figure 3h).

### Peptide immunizations modulate tumor microenvironment specifically affecting mutated IDH1 expression

**Figure 4:**
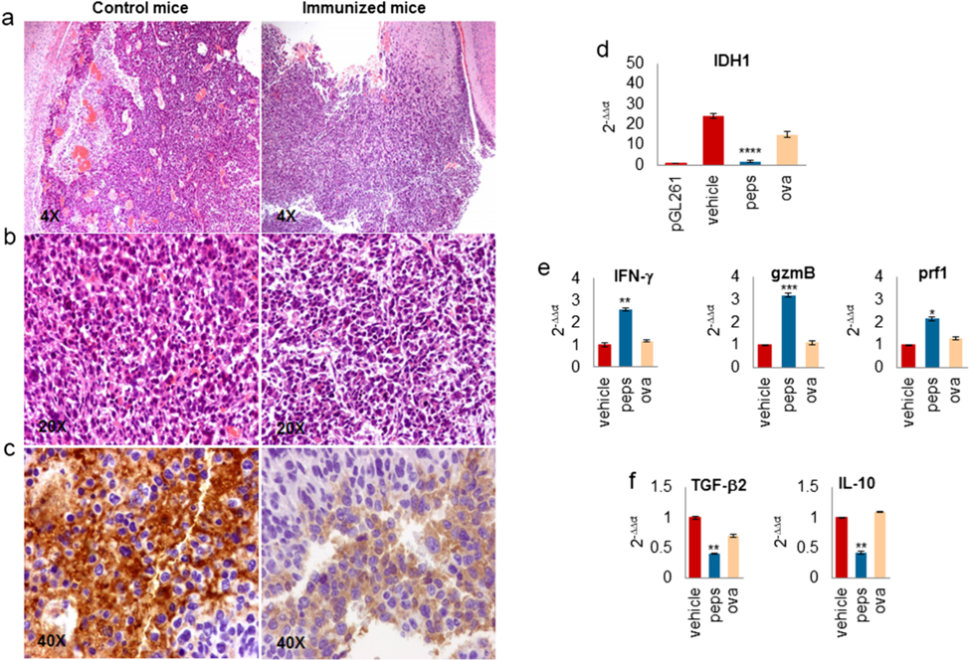
**Immunization with peptides targets IDH1-R132H and modulates the tumor microenvironment. a**, **b** H&E of representative mIDH1-GL261 gliomas from vehicle and immunized mice confirms a strong vascularization an a higher presence of blood vessels in gliomas from mice treated with vehicle. **c** Gliomas from immunized mice show a lower cell density and absence of mIDH1 expression. **d** RT-PCR shows IDH1 expression in gliomas from control mice and a significant reduced expression in immunized mice. The relative expression of IDH1 was normalized with that detected in gliomas from pGL261. **e**, **f** RT-PCR on explanted gliomas shows that IFN-γ, granzyme (gzm)-b and perforin (prf)-1 were expressed at higher levels, TGF-β2 and IL-10 expression at lower levels in gliomas from immunized mice than controls. Data reported as peps are mean ± SD of the two groups of immunized mice.

Gliomas from mice treated with vehicle maintained highly vascularized features showing large and medium size-vessels. In gliomas from immunized mice the number of blood vessels was reduced (Figure 4a, b) and was similar to pGL261-gliomas (Additional file 2: Figure S1d). HIF-1α and VEGF expression were significantly reduced in gliomas from immunized mice compared with controls (Additional file 2: Figure S3).

Vehicle gliomas appeared immune-reactive for IDH1-R132H, whereas gliomas from immunized mice almost completely lost IDH1-R132H expression (Figure 4c), as also confirmed by RT-PCR (Figure 4d).

The immune response induced by peptide immunizations determined a significant modulation of tumor microenvironment. IFN-γ, granzyme-b and perforin-1 expression levels were 2.6 ± 0.04, 3.2 ± 0.03- and 2.1 ± 0.02- fold higher, respectively, in immunized mice than in vehicle mice (*p* < 0.01, *p* < 0.005, *p* = 0.01; Figure 4g). In contrast, TGF-β2 and IL-10 expression levels were 2.4 ± 0.22- and 2.3 ± 0.01-fold lower, respectively, in immunized mice than in control animals (*p* = 0.01 and *p* < 0.005; Figure 4h).

## Discussion

Recent data have shown that the most frequent IDH1 mutation, R132H, can be effectively targeted in a A2.DR1 mice devoid of mouse MHC and transgenic for human MHC class I and II [14]. The R132H mutation was expressed in sarcoma cells obtained by exposure to 3-methylcholantrene that were injected subcutaneously in the A2.DR1 mice [14]. Our results show for the first time in an intracranial glioma model that the R132H mutation, can be effectively targeted by the immune system, allowing a significant prolongation of survival and the cure of 25% of the mice. The mIDH1-GL261 model we have used has obvious differences with human gliomas, as it is established in a malignant glioma while in humans IDH1 mutations take place early in development of low grade gliomas. An important similarity, however, is that, as in the human counterpart, mIDH1-GL261 cells produce increased amounts of the oncometabolite 2HG in vitro and in vivo. Although at a much lower level, pGL261 also release detectable amounts of 2HG. As mentioned in Results, levels higher than background were found in a subset of wild type IDH gliomas [22]. Interestingly, 2HG production was also found in breast cancer cells in association to MYC overexpression [26]. This may also be the case of pGL261 cells, as they show increased myc expression [27]. The presence of the mutation is associated to a decreased growth in vivo, exemplified by MRI scans taken on day 8 and 20 after intracranial injection of tumor cells (Figure 1g, h) and by the trend in Kaplan Meier survival curves until day 28 (Figure 1f), even if differences with pGL261 were not statistically significant. A recent research by Phillips and colleagues may provide some explanation for this finding, as it shows that IDH1-R132H exhibits a growth-inhibitory effect (possibly caused by deficiency in metabolic flux from glucose and glutamine to lipids) that is abrogated in the presence of the hominoid-specific enzyme glutamate dehydrogenase 2 (GLUD2) [28].

Overall, however, the lethality of pGL261 and mIDH1-GL261 used in our experiments, was similar. This could be partly due to the progressive decrease of the fraction of mIDH1-GL261 cells in vivo*,* as suggested by decreased 2HG levels detected on day 20 vs day 8 (Figure 2).

Of interest is the increased vascularization and the hemorrhagic features of mIDH1-GL261 gliomas. Initial data suggested that reduced formation of alpha-ketoglutarate (alpha-KG) may increase the levels of hypoxia-inducible factor subunit HIF-1α [10]. Subsequent studies had difficulties in replicating those findings [29,30]. Notably, the brain-specific knock-in of the R132H mutation was associated to hemorrhages in the presence of increased HIF-1α expression and deficient collagen maturation and basement membrane function [23].

Despite the low score obtained using the class I MHC binding prediction we immunized immune competent IDH1 mutated glioma-bearing mice using four short peptides (two 9-mers and two 10-mers). A CD8+ T cell response, specific cytotoxicity and an antibody response were activated leading to a significant increase of survival. In particular, specificity was suggested by the lack of immune response when peptide vaccination took place in mice bearing parental GL261, lacking the IDH1 mutation. Immunization with the 16-mer peptide containing MHC class I and class II epitopes only induced a CD8+ T cells without antibody response. Survival is significantly higher than controls but less than what obtained with short peptide immunizations, possibly because of the lack of activation of the antibody response.

CTL epitopes alone can be inefficient in inducing a long-term immune response and a specific memory [31], whereas MHC class I and class II restricted epitopes in a single longer peptide can improve vaccine efficacy based on the simultaneous activation of CTL and CD4+ T cells [32-34]. This condition was observed after immunizations with 35 amino-acid long peptides containing CTL epitopes showed more efficient than those with CTL peptides in inducing effective anti-tumor cytotoxic response [35].

A number of reports support the essential role of CD4+ T cells in the anti-tumor responses due to their ability to stimulate dendritic cells and potentiate anti-tumor immune response by enhancing antigen presentation [36,37] and to the contribution to the memory response establishment [38]. Importantly CD4+ T cells have also been described to develop cytotoxicity and they are able to eradicate established melanomas [39]. However there is also evidence that long peptides can modulate the efficacy of immunization by affecting the magnitude of CTL response [40,41].

In our experiments we used as immune adjuvants GM-CSF and Montanide ISA-51, a water-in-oil emulsion enhancing the cytotoxic CD8+ T lymphocyte response. This was reported in patients [42-44], and was also supported by our previous pre-clinical experiments, where immunizations with short peptides induced CD8+ T cell positive activation and tumor specific cytotoxicity [17,18].

In conclusion, both our data and those of Platten and coworkers [14] converge in supporting a translational evolution of immunological targeting of the R132H mutation of IDH1 in glioma patients.

There is increasing clinical evidence that immune checkpoint inhibitors like ipilimumab and nivolumab may prolong survival in patients with melanoma and small cell lung cancer [45,46]. Preclinical evidence support a role for this therapeutic approach in gliomas [47] and a clinical trial is now ongoing to test this hypothesis. It is plausible that a combination of this nonspecific form of activation of the immune system with specific mutation targeting, as is the case for the R132H mutation, may yield a synergic effect. This hypothesis deserves further testing in an appropriate murine model.

## Additional files

Additional file 1:
**Supplementary methods.**
Additional file 2: Figure S1. mIDH1-GL261 gliomas maintain 2HG production and are strongly hemorrhagic. **Figure S2.** Splenocytes from mice immunized with IDH1-R132H peptides do not lyse pGL261 cells. **Figure S3.** Gliomas from immunized mice show a decreased expression of HIF1-α and VEGF.
