# Supplementary material for: Effective immuno-targeting of the IDH1 mutation R132H in a murine model of intracranial glioma
Source: Acta Neuropathol Commun. 2015 Jan 21;3:4. doi: 10.1186/s40478-014-0180-0 (PMC4359524; doi:10.1186/s40478-014-0180-0)
Supplement: Additional file 1: — Supplementary methods. [file 40478_2014_180_MOESM1_ESM.pdf]

Additional File 1

Site-directed mutagenesis

Internal mutagenic oligonucleotides (FW - I- ATC ATC ATT GGC CAT CAT GCA TAT GGG GAC; RV -I-TAG TAG TAA CCG GTA GTA CGT ATA CCC CTG) were used to convert the CGA codon read as Arginine in CAT codon read as histidine.

External oligonucleotides were used to introduce enzymatic restriction site on the mIDH1 cDNA (EcoRI – FW – CGG AAT TCA TGT CCA GAA AAA TCC AAG GAG; Xba - RV- GCT CTA GAT TAA AGT TTG GCC TGA GCT AAT). Three PCR were performed; the first using F_I and Xba-R oligonucleotides, the second using Eco-F and R-I oligonucleotides. The two templates were combined to obtain PCR mutant product, using EcoRI-F and XbaI-Roligonucleotides. The Finnzyme Phusion High-fidelity DNA Polimerase system was used for every amplification using the following profile: starting denaturation (98°C, 30 sec), denaturation (98°C, 10 sec), annealing (68°C, 30 sec), and extension (72 °C, 30 sec), final extension (72 °C, 10 min). The amplified product was cloned into pCMV6kan-neo vector after the enzymatic digestion, and sequenced.

Oligo sequences

IFN-γ:

FW-5’-ATCTGGAGGAACTGGCAAAA-3’

RV-5’-TTCAAGACTTCAAAGAGTCTGAGGTA-3’

TGF-β2:

FW-5’-TGGAGTTCAGACACTCAACAC-3’

RV-5’-AAGCTTCGGGATTTATGGTG-3’

Granzyme-b:

FW-5’-GCTGCTCACTGTGAAGGAAGT-3’

RV-5’-TGGGGAATGCATTTTACCAT-3’

IL-10:

FW-5’-CAGAGCCACATGCTCCTAGA-3’

RV-5’-GTCCAGCTGGTCCTTTGTTT-3’

Perforin-1:

FW-5’-TGAGAAGACCTATCAGGACCAG-3’

RV-5’-GTCAAGGTGGAGTGGAGGTT-3’

IDH1:

FW-5’-AGACTCAGTCGCCCAAGGT-3’

RV-5’-GCGGTAGTGACGTGTGACAG-3’

beta-actin:

FW-5’-GATGTGGATCAGCAAGCAGGA-3’

RV-5’-AGCTCAGTAACAGTCCGCCTA-3’
