# Supplementary material for: Effective immuno-targeting of the IDH1 mutation R132H in a murine model of intracranial glioma
Source: Acta Neuropathol Commun. 2015 Jan 21;3:4. doi: 10.1186/s40478-014-0180-0 (PMC4359524; doi:10.1186/s40478-014-0180-0)
Supplement: Additional file 2: Figure S1. — mIDH1-GL261 gliomas maintain 2HG production and are strongly hemorrhagic. Figure S2. Splenocytes from mice immunized with IDH1-R132H peptides do not lyse pGL261 cells. Figure S3. Gliomas from immunized mice show a decreased expression of HIF1-α and VEGF. [file 40478_2014_180_MOESM2_ESM.pdf]

Additional File 2

Figure S1. mIDH1-GL261 gliomas maintain 2HG production and are strongly hemorrhagic

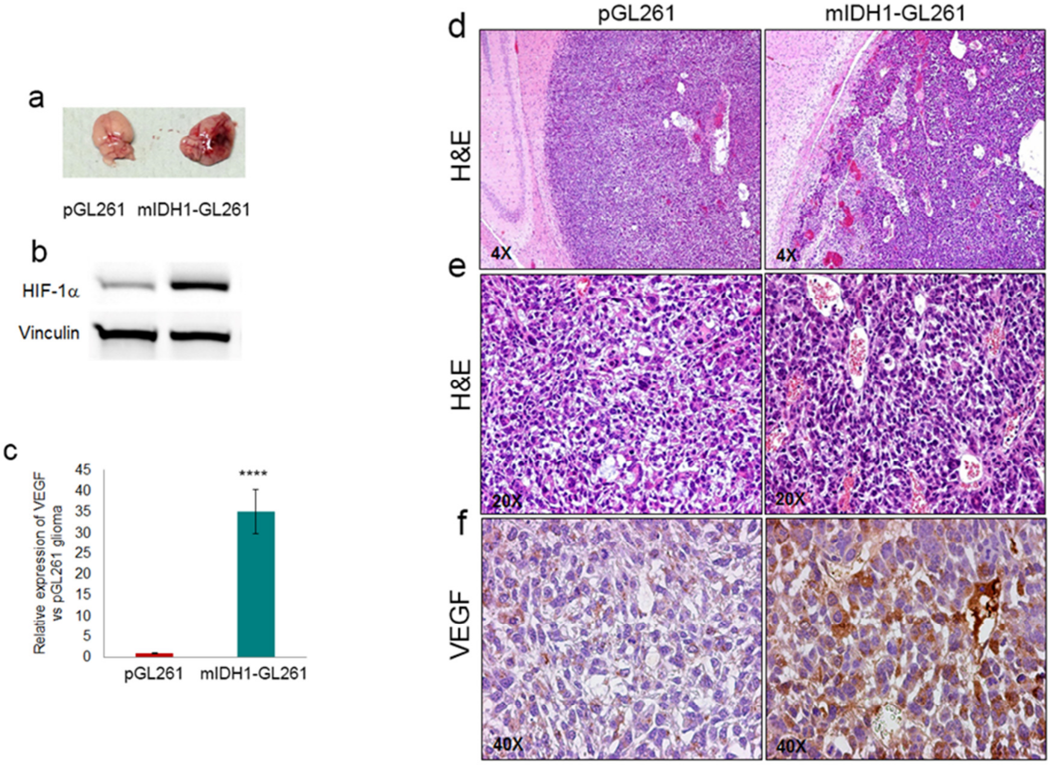

a Representative images of brains explanted on day 27 after implantation show that mIDH1-GL261 glioma were highly bloody compared with control. **b** Western blot analysis of HIF-1α expression on explanted mIDH1-GL261 gliomas shows an increased expression compared to controls. Vinculin was used as housekeeping protein. **c** RT-PCR performed on the same explanted gliomas reveals an increased expression of VEGF in mIDH1-gliomas compared to controls. **d, e** H&E of representative gliomas shows that mIDH1-GL261 gliomas were more vascularized than controls. **f** Histology on the same gliomas confirmed high expression levels of VEGF in mIDH1-GL261 gliomas.

Figure S2. Splenocytes from mice immunized with IDH1-R132H peptides do not lyse pGL261 cells.

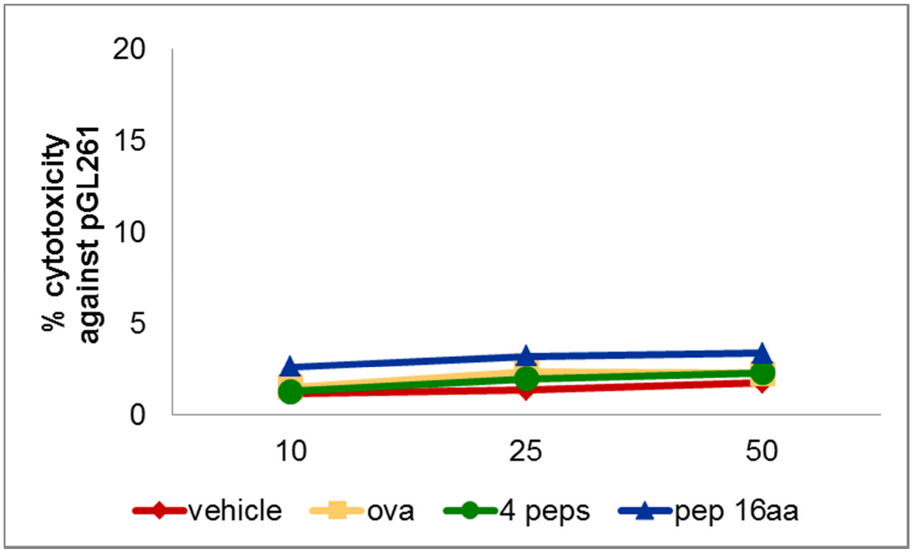

In vitro MTT assay reveals that splenocytes from immunized mice do not recognize pGL261 as target cells as well as splenocytes from vehicle and ova control mice.

Figure S3. Gliomas from immunized mice show a decreased expression of HIF1-α and VEGF

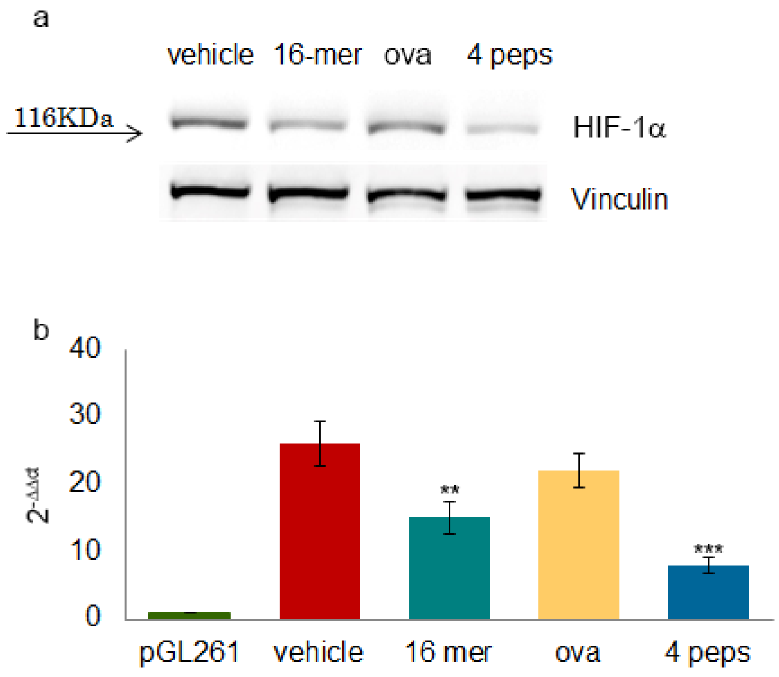

a Western blot analysis performed on explanted gliomas shows a decreased expression of HIF-1α in immunized mice compared to controls. Vinculin was used as housekeeping protein. **b** RT-PCR indicates a significant reduced expression of VEGF expression in immunized mice compared to controls.
